# Yeast (*Saccharomyces cerevisiae*) GC/EI/MS metabolomics dataset

**DOI:** 10.1016/j.dib.2020.105208

**Published:** 2020-01-30

**Authors:** Dimitra A. Karamanou, Konstantinos A. Aliferis

**Affiliations:** a Laboratory of Pesticide Science, Agricultural University of Athens, Iera Odos 75, 118 55, Athens, Greece; b Department of Plant Science, McGill University, 21111 Lakeshore Road, Sainte-Anne-de-Bellevue, Quebec, H9X 3V9, Canada

**Keywords:** Fungal resistance, Plant protection products, Transporters, Yeast metabolomics

## Abstract

Gas chromatography-electron impact-mass spectrometry (GC/EI/MS) global profiling of the endo-metabolome of wild and genetically engineered yeast (*Saccharomyces cerevisiae*) strains was performed. The strains were treated or not with sub-lethal doses of the fungicide flusilazole, in order to mine the involvement of the ABC transporter YCF1, which is located in the yeast vacuole membrane, in its resistance to fungicides. Raw GC/EI/MS total ion chromatograms (*.cdf format) of the yeast endo-metabolome were recoded, which are included in this article. Since yeast is a model organism, the dataset could serve as a reference for yeast metabolomics studies related to the investigation of the effects of bioactive ingredients on its metabolism. The dataset support the research article “*Karamanou D. and Aliferis K.A, 2019. The yeast (Saccharomyces cerevisiae) YCF1 vacuole transporter: evidence on its implication into the yeast resistance to flusilazole as revealed by GC/EI/MS metabolomics. Pest. Biochem. Physiol.* doi: https://doi.org/10.1016/j.pestbp.2019.09.013”. 10.1016/j.pestbp.2019.09.013.

**Table:** Specifications Table

Subject	Agricultural and Biological Sciences (General)
Specific subject area	Plant protection product (PPPs) research and development-study of the mechanisms of fungal resistance to PPPs
Type of data	Raw GC/EI/MS total ion chromatograms (*.cdf format)
How data were acquired	Untargeted GC/EI/MS metabolomics analysis Instrument: Agilent 6890 MS platform (Agilent Technologies Inc.), equipped with a 5973 series mass selective detector (MSD) and a 7683 autosampler Acquisition of data using the MSD Chemstation (Agilent)
Data format	Raw (*.cdf)
Parameters for data collection	Column: HP-5MS, length; 30 m, i.d.; 0.25 mm, film thickness 0.25 μm, Agilent Technologies Inc. Split ratio: 5:1 Injector temperature: 230 °C Oven temperature: 70 °C, stable for 5 min, 5 °C min^−1^ increase to 295 °C, stable for 2 min. Carrier gas: Helium, 1 mL min^−1^ flow rate Ionization: Positive electron ionization, 70eV Full scan 50–800 Da (4 scans s^−1^) Temperature of the MS source, 230 °C, quadrupole 230 °C
Description of data collection	TIC of the yeast end-metabolomes performing full scanning over the mass range between 50 and 800 Da
Data source location	Agricultural University of Athens Athens Greece
Data accessibility	Repository name: Pesticide Metabolomics Group database Data identification number: *Yeast (Saccharomyces cerevisiae) (PMG-03-19)* Direct URL to data: https://www.aua.gr/pesticide-metabolomicsgroup/Resources/default.html
Related research article	*D. Karamanou and K.A. Aliferis* Title; *The yeast (Saccharomyces cerevisiae) YCF1 vacuole transporter: evidence on its implication into the yeast resistance to flusilazole as revealed by GC/EI/MS metabolomics.* Journal; *Pest. Biochem. Physiol.* DOI: https://doi.org/10.1016/j.pestbp.2019.09.013

**Table:** 

**Value of the Data** • The data provide an overview of the effects of the triazole fungicide flusilazole on the metabolism of yeast • Data could be used by researchers working on the investigation of the mechanisms by which microorganisms develop resistance to bioactive compounds • Data provide new insights into the involvement of the YCF1 transporter in the development of fungal resistance to fungicides • To the best of our knowledge, no similar data exist on the effect of the YCF1 transporter on the metabolism of yeast and its resistance to triazoles

## 1 Data

The data set includes raw total ion chromatograms (TIC) of yeast (*Saccharomyces cerevisiae*) endo-metabolomes in “*.cdf” format. Untargeted GC/EI/MS analysis was performed. The TIC correspond to profiles of untreated wild (*WC*) and genetically engineered (*YC*) yeast strains and respective flusilazole-treated WF and YF strains. Analyses were performed 39 h following treatments. Incubation of the cultures was performed in an orbital incubator at 30 °C under constant shaking (250 rpm), in the dark.

## 2 Experimental design, materials, and methods

The isogenic yeast strains *Wt* and *Δycf1* were used for the dissection of the involvement of the vacuole YCF1 transporter in yeast resistance to flusilazole applying a functional genomics approach [1]. The preparation of the samples, including the quenching of cell metabolism and cell washing was performed following a 2-step addition of cold methanol (−32 °C) [2].

For the extraction of the yeast endo-metabolome, the organic solvents ethyl acetate and methanol (MeOH) (GC/MS grade, 99.9% purity) (Carlo Erba Reagents, val de Reuil, France) were used. In the sample preparation for GC/EI/MS metabolomics pyridine (99.8%, v/v), methoxylamine hydrochloride (98%, w/w), ribitol, and analytical standards of yeast metabolites, were used (Sigma-Aldrich Ltd., Darmstadt, Germany). For the silylation of samples, *N*- Trimethylsilyl-*N*-methyl trifluoroacetamide (MSTFA, Macherey and Nagel, Düren, Germany), was used.

The derivatized extracts (1 μL) were injected on column (HP-5MS, length; 30 m, i.d.; 0.25 mm, film thickness 0.25 μm, Agilent Technologies Inc.) applying a 5:1 split, at an injector temperature of 230 °C. The temperature of the oven was set initially at 70 °C, kept stable for 5 min, followed by a 5 °C min^−1^ increase to 295 °C, kept stable for 2 min. Helium was the carrier gas at 1 mL min^−1^ flow rate. Positive electron ionization at 70eV was applied and full scan mass spectra were acquired over the mass range of 50–800 Da (2 scans s^−1^), with a 10 min solvent delay. The temperature of the MS source was set at 230 °C and that of the quadrupole at 230 °C.

The acquired GC/EI/MS metabolite profiles were initially deconvoluted using the software AMDIS v.2.66 (NIST; Gaithersburg, MD, USA). The GC/EI/MS data pre-processing for metabolomics analyses was performed as previously described pipeline [3]. The discovery of trends within the obtained dataset and the discovery of the biomarkers of flusilazole toxicity to *S. cerevisiae*, was based on multivariate analyses [4,5].
